# Epstein-Barr Virus and Human Papillomavirus Infection in Papillary Thyroid Carcinoma: A Correlation Study

**DOI:** 10.2174/0118715303355722250127061453

**Published:** 2025-03-10

**Authors:** Runyu Zhao, Yingying Lu, Weiqiang Teng, Xiaocheng Xue, Yi Zhang, Shuixian Huang, Xiaoping Chen

**Affiliations:** 1 Postgraduate Training Base at Shanghai Gongli Hospital, Ningxia Medical University, Shanghai 200135, China;; 2 Shanghai Health Commission Key Lab of Artificial Intelligence (AI)-Based Management of Inflammation and Chronic Diseases, Department of Central Laboratory, Gongli Hospital of Shanghai Pudong New Area, Shanghai 200135, China;; 3 School of Medicine, Shanghai University, Shanghai 200444, China;; 4 Department of Otolaryngology, First Affiliated Hospital of Soochow University, Suzhou 215000, China;; 5 Department of Otolaryngology, Gongli Hospital of Shanghai Pudong New Area, Shanghai 200135, China

**Keywords:** Epstein-Barr Virus, Human Papillomavirus, papillary thyroid carcinoma, Hashimoto's thyroiditis, pathogenic mechanisms, thyroid cancer

## Abstract

**Introduction:**

This study explores the presence and clinical significance of Epstein-Barr Virus (EBV) and Human Papillomavirus (HPV) in papillary thyroid carcinoma (PTC). EBV and HPV are known to contribute to various cancers, but their roles in thyroid cancer development are debated.

**Methods:**

Paraffin-embedded tissue blocks from PTC patients (n=255) who underwent thyroid surgery between 2020 and 2021 were analyzed for EBV and HPV DNA using PCR-based methods.

**Results:**

Results showed EBV positivity in 45.1% of PTC cases, significantly higher than in benign thyroid tumors (35.2%), while HPV positivity was low (0.7%). EBV positivity was not associated with age, gender, lesion type, lymph node metastasis, or extrathyroidal extension but was significantly higher in PTC cases with concurrent Hashimoto's thyroiditis. The study suggests a notable presence of EBV in PTC, especially in cases with Hashimoto's thyroiditis, but indicates a limited role for HPV in thyroid cancer.

**Conclusion:**

Further research is warranted to understand the specific pathogenic mechanisms and potential therapeutic implications of EBV and HPV in thyroid cancer.

## 1 INTRODUCTION

The Epstein-Barr Virus (EBV), formally known as Human Herpes Virus 4, belongs to the herpes family and is one of the nine known human herpesvirus types. It stands out as the most widespread and growth-prone human pathogen [1, 2]. EBV exhibits a high prevalence in the population, particularly among children and adolescents, with approximately 95% of the global population testing positive for EBV serum (IgG) [3]. EBV has been confirmed to be closely associated with various malignant tumors, including nasopharyngeal carcinoma, gastric cancer, Hodgkin's lymphoma, and Burkitt lymphoma, among others [2, 4-6], and can induce tumor formation and progression. Upon infecting host cells, EBV initiates the expression of different genes through lytic or latent infection, including latent membrane proteins (such as LMP1, LMP2A, and LMP2B), EBV nuclear antigens (including EBNA1, EBNA2B, *etc.*), and two small RNAs (EBER-1 and EBER-2) [7]. By causing systemic inflammation, inhibiting the anti-tumor immune system, and promoting resistance to apoptosis, EBV induces tumor development [8, 9].

Human Papillomavirus (HPV) is a double-stranded DNA virus that infects epithelial cells, primarily of the skin and mucous membranes [10]. It is the most common sexually transmitted infection globally, with over 200 different genotypes identified to date [11]. While most HPV infections are transient and asymptomatic, persistent infections with high-risk HPV genotypes, notably HPV-16 and HPV-18, are strongly associated with the development of various cancers [12]. The oncogenic potential of HPV lies in its ability to disrupt cellular regulatory mechanisms, leading to uncontrolled cell proliferation and eventual malignant transformation [13]. Understanding the relationship between HPV infection and cancer development is crucial for implementing effective preventive strategies, such as HPV vaccination and screening programs, to reduce the burden of HPV-associated cancers [14].

Thyroid cancer is the most common endocrine malignancy, accounting for approximately 2.1% of all global cancers, with papillary thyroid cancer (PTC) as the most common pathological type of thyroid cancer [15]. In recent decades, the incidence of thyroid cancer has rapidly increased worldwide, reaching approximately 580,000 new cases globally in 2020, making it the 9th most common cancer [16]. This underscores the importance of identifying new risk factors associated with thyroid tumor occurrence and development. However, the role of EBV and HPV in thyroid infection and its involvement in the progression of thyroid cancer remains controversial.

Therefore, our study retrospectively investigates the role of EBV in the development of PTC within a cohort and explores its clinical significance.

## 2 MATERIALS AND METHODS

### 2.1 Tissue Sample

Paraffin-embedded tissue blocks from all patients with PTC who underwent thyroid surgery at our hospital between 2020 and 2021 were collected, and pathology report information was evaluated. Based on the final pathology report after thyroidectomy, 255 patients diagnosed with PTC and 179 patients with benign tumor were included. The metastatic focus of PTC in other tissues (for example, lymph node) was not used. No other inclusion and exclusion criteria were used. This study was approved by the ethics committee of our institution (Approval No. 2020 Research Review No. 69). All 434 patients signed the written informed consent.

### 2.2 DNA Extraction

DNA from tissue samples was extracted using a DNA extraction kit (Hybribio, China). Tissue samples embedded in paraffin blocks were microdissected and transferred to sterile tubes with the addition of xylene. After deparaffinization at room temperature, the supernatant was centrifuged and discarded, and ethanol was added to hydrate the samples. Sodium dodecyl sulfate and proteinase K were added, followed by overnight incubation at 56°C. After centrifugation with the addition of isopropanol, DNA was extracted using TE elution buffer.

### 2.3 EBV DNA Detection

The EBV DNA was detected using the PCR-fluorescent probe method. According to GenBank EBV DNA segment, the following primer sequences for HPV DNA amplification were designed: upstream primer, 5’-CCAGAGGTAAGTGGACTTTA-3’; and downstream primer, 5’-CCTTCTTAGGAGCTGTCC-3’. The TaqMan fluorescent probe sequence (5’FAM-TAAGCCCAACACTCCACCAC-BHQ3’) was labeled with reporter dye at the 5’-end and quencher dye at the 3’-end [17].

PCR was carried out in a 50 µl reaction, which included 10 μl of template DNA and 40 μl of pre-prepared reaction solution. The template DNA included positive samples, negative samples, quantitative reference standards, and test samples. The reaction solution contained primers, probes, dNTPs, DNA polymerase, uracil-DNA glycosylase, *etc.* The reaction system was first treated at 50°C for 2 minutes, followed by a 5-minute treatment at 94°C, and then subjected to 45 cycles of PCR amplification. The temperature cycle involved a first step at 94°C for 15 seconds and a second step at 57°C for 30 seconds. For samples with a Ct value ≤39, the result was reported as EBV-positive. For samples with Ct >39, if the internal control detection was positive, the report indicated that the EBV DNA was below the detection kit's lower limit. For Ct >40, the detection result was considered invalid.

### 2.4 HPV DNA Detection

HPV was detected using a kit for the detection of 37 HPV genotypes (PCR + reverse dot blot hybridization method) (Hybribio, China). DNA samples were subjected to PCR amplification, and the Kap Medical Nucleic Acid Molecular Rapid Hybridization Instrument was used as a platform for reverse dot blot hybridization to detect the hybridization results with type-specific probes. Clear blue-purple dots were interpreted as positive.

### 2.5 Statistical Analysis

Chi-square was used to compare the EBV gene frequency between the groups. Statistical analyses were performed using SPSS software v.23 for Windows (SPSS Inc., Chicago, IL), and a *P* value < 0.05 was considered statistically significant.

## 3 RESULTS

### 3.1 HPV and EBV Infection in PTC

**Fig. (1):**
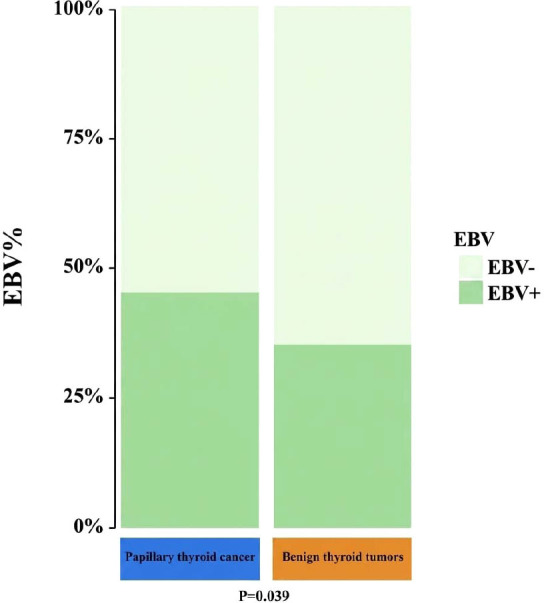
Prevalence of Epstein-Barr virus (EBV) in patients with thyroid tumors. EBV is more prevalent in patients with papillary thyroid cancer than in patients with benign thyroid tumors (*P* = 0.039).

Among 255 cases of PTC and 179 cases of benign thyroid tumors, EBV positivity was observed in 115 cases (45.1%) of PTC and 63 cases (35.2%) of benign thyroid tumors, showing a statistically significant difference between the two groups (*P* = 0.039, Fig. **1**). Among 150 samples of PTC and 133 samples of benign thyroid tumor subjected to HPV testing, only one PTC case (0.7%) tested positive, showing HPV 11 subtype expression.

### 3.2 The Relationship between EBV Infection and Clinical Pathological Characteristics in PTC Samples

As shown in Table **1**, we compared the clinical and pathological characteristics between patients with EBV-positive and negative PTC. The average age (mean ± standard deviation) of EBV-positive and negative patients was 44 ± 12 years and 43 ± 12 years, respectively (*P* = 0.455). Additionally, there were no significant differences between EBV-positive and negative patients in terms of gender, lesion type, lymph node metastasis (LNM), and extrathyroidal extension (ETE). However, among PTC patients with coexisting Hashimoto's thyroiditis, the EBV positivity rate (62.3%) was significantly higher than that of PTC patients without Hashimoto's thyroiditis (38.7%).

## 4 DISCUSSION

In recent years, there has been limited research on the association between EBV and thyroid cancer, and controversy exists. In our collected samples of PTC, 45.1% tested positive for EBV, which is higher than the EBV positivity observed in the control group of benign thyroid tumors. Additionally, 0.7% of PTC samples tested positive for HPV, while all collected benign thyroid tumor samples tested negative for HPV. This suggests that EBV may play a role in the occurrence and development of PTC, while the involvement of HPV in PTC development remains questionable. However, our results indicate some discrepancies compared to other reported studies.

Stamatiou *et al.* detected EBV segment expression in 30 thyroid cancer or multinodular goiter tissues using PCR, finding a positive expression in 90% (27/30) of cases [18]. Almeida *et al.,* analyzing 183 thyroid samples (including 100 malignant tumors and 83 benign tumors) through PCR, discovered a positive expression in 16% (29/183) of the samples, with higher viral loads in malignant tumors compared to benign or normal tissues [19]. Homayouni *et al.* detected EBV segments in PTC tissues using PCR, with a positivity rate of 65.8% (27/41) [20]. Another study collected 57 thyroid cancer samples and 18 healthy control samples, detecting EBV segment expression through PCR, and found a positive expression in 71.9% (29/57) of the cancer samples [8]. In contrast, Bychkov *et al.* analyzed EBV expression in 20 thyroid cancer samples using in situ hybridization and immunohistochemical staining, finding no positive expression [21]. Yu *et al.* collected 384 thyroid cancer samples and detected EBV EBER expression using in situ hybridization, finding all samples to be negative [9]. The significant variation in the positive expression rates of EBV in thyroid cancer among different studies may be attributed to geographical factors, detection methods, and other variables.

Despite the considerable differences in the detection rates of EBV in thyroid cancer, some studies have explored potential mechanisms of EBV in thyroid cancer. Shimakage *et al.* observed that the signal intensity of EBV DNA in situ hybridization was higher in undifferentiated thyroid cancer compared to PTC, suggesting that EBV may promote dedifferentiation of thyroid tumors [22]. Almeida *et al.* conducted the first study using EBV-infected thyroid cancer cell lines, finding that all tumor cell lines could be infected with EBV, and EBV infection could interfere with the expression of oncogenes, although with variations between different cell lines [23]. This suggests a potential role of EBV in tumor progression, but the specific mechanisms remain unclear. Moghoofei *et al.* found that EBV-encoded products could upregulate the expression of certain genes (anti-apoptotic protein survivin, CD44, NF-κB, and Bcl-2), contributing to anti-apoptosis and promoting the progression of thyroid cancer [8]. Additionally, another study by Moghoofei *et al.* proposed that EBV might contribute to thyroid cancer development through chronic inflammation [24]. Xie *et al.,* through bioinformatic analysis, explored potential mechanisms causing immune differences in PTC, identifying EBV infection as the most significantly enriched pathway in the Kyoto Encyclopedia of Genes and Genomes (KEGG), suggesting a potential role of EBV in the changes of the immune microenvironment in thyroid cancer [25]. However, further research is needed to investigate how EBV specifically causes changes in the immune microenvironment of thyroid cancer. Given that lymphocytes are the primary reservoir for the virus, and infiltrating lymphocytes are common in thyroid cancer, which might be the main reservoir for EBV [26, 27]. Based on this, Almeida *et al.* hypothesized that EBV may not be the cause of thyroid cancer development but could be a bridge linking autoimmune thyroiditis and thyroid cancer, serving as one of the exogenous factors promoting their growth and evolution [28]. It is noteworthy that a study found 80.7% (21/26) of Hashimoto's thyroiditis samples to be EBV-positive, suggesting a potential role of EBV in autoimmune thyroiditis [29]. Another study found that in cases of PTC developing on a background of Hashimoto's thyroiditis, EBV was positively detected in both tumor tissues and normal follicular cells. This suggests a potential link between Hashimoto's thyroiditis and EBV [30]. In our study, we found a higher EBV positivity rate in PTC patients with coexisting Hashimoto's thyroiditis. The role of EBV in autoimmune thyroiditis and thyroid cancer still needs further exploration.

Stamatiou *et al.* detected HPV DNA in surgical samples collected from 30 patients with thyroid cancer or thyroid nodules, yet no samples tested positive [31]. Dialameh *et al.* collected 82 samples of PTC and 77 samples of thyroid nodules for PCR testing, revealing HPV PCR positivity in 3.8% of benign thyroid nodules and 13.4% of PTC specimens [32]. The prevalence of HPV PCR positivity in PTC tissue was significantly higher than in benign thyroid nodules, suggesting a notable association between PTC and HPV [32]. Another study investigating the history of past HPV infections in thyroid cancer patients found a higher prevalence of past HPV infections among thyroid cancer patients compared to the control group, indicating a significant link between past HPV infections and thyroid cancer development [33]. However, our study only identified 0.7% of PTC cases with HPV DNA positivity, thus precluding a conclusive association between HPV and PTC. Overall, research on the correlation between thyroid cancer and HPV infection is limited, and further investigation is warranted to elucidate their relationship.

Despite significant regional, population, and methodological variations in the detection rates of EBV and HPV in thyroid samples, the mechanisms influencing the occurrence and development of thyroid cancer are gradually being revealed. Currently, the role of EBV and HPV in thyroid cancer is not fully understood, and its impact on the prognosis of thyroid cancer remains unclear. Therefore, there is an urgent need for larger sample sizes and more reliable detection methods for further in-depth research. This will help better understand the oncogenic mechanisms of EBV, potentially reducing the occurrence of thyroid cancer and finding more tailored treatment options to improve the prognosis of thyroid cancer patients.

## 5 LIMITATIONS

This study has several limitations. First, the sample size is relatively small, which may affect the generalizability of the findings. Second, this study is observational in nature and does not establish a causal relationship between EBV/HPV infection and PTC development. Further mechanistic studies are needed to explore the underlying biological pathways. Lastly, the detection of EBV and HPV was primarily based on molecular techniques, and additional validation using in situ hybridization or immunohistochemical analysis could strengthen the results. 

## CONCLUSION

In conclusion, this study indicates a certain proportion of EBV infection in PTC, while the HPV infection rate is lower. These findings indicate that EBV may play a role in the development and progression of PTC, whereas the role of HPV in PTC appears to be limited. Moreover, PTC tissues with concurrent Hashimoto's thyroiditis are more prone to EBV infection compared to those without Hashimoto's thyroiditis. However, further in-depth research is needed to elucidate the specific pathogenic mechanisms following infection.

## Figures and Tables

**Table 1:** Correlation between EBV and clinicopathological features of PTC.

**Parameter**	-	**EBV Positive N (%)**	**EBV Negative N (%)**	***P* value**
**Gender**	Male	27 (39.7)	41 (60.3)	0.297
	Female	88 (47.1)	99 (52.9)	
**Tumor Diameter**	≤1cm	85 (44.7)	105 (55.3)	0.459
	1~2cm	26 (47.3)	29 (52.7)	
	2~4cm	4 (50.0)	4 (50.0)	
	>4cm	0 (0.0)	2 (100.0)	
**LNM**	Yes	4 (26.7)	11 (73.3)	0.463
	No	111 (46.3)	129 (53.8)	
**TNM stage**	I	103 (41.0)	148 (59.0)	0.150
	II	0 (0.0)	4 (100.0)	
**Location**	Isthmus	2 (66.7)	1 (33.3)	0.359
	Unilateral	104 (43.9)	133 (56.1)	
	Bilateral	9 (60.0)	6 (40.0)	
**Focus type**	Unifocal	88 (43.3)	115 (56.7)	0.268
	Multifocal	27 (51.9)	25 (48.1)	
**ETE**	Yes	2 (28.6)	5 (71.4)	0.373
	No	113 (45.6)	135 (54.4)	
**Hashimoto**	Yes	43 (62.3)	26 (37.7)	0.001*
	No	72 (38.7)	114 (61.3)	
**Total**	-	115 (45.1)	140 (54.9)	-

**Note:** ETE= Extrathyroid extension, LNM= lymph node metastasis, * *P* < 0.05.

## Data Availability

All the data and supporting information are provided within the article.

## LIST OF ABBREVIATIONS

EBV: Epstein-Barr Virus
HPV: Human Papillomavirus
PTC: Papillary Thyroid Carcinoma
LNM: Lymph Node Metastasis
ETE: Extrathyroidal Extension

## STANDARDS OF REPORTING

STROBE guidelines were followed.
